# Supplementary material for: Vaginal Lactobacilli and Vaginal Dysbiosis-Associated Bacteria Differently Affect Cervical Epithelial and Immune Homeostasis and Anti-Viral Defenses
Source: Int J Mol Sci. 2021 Jun 17;22(12):6487. doi: 10.3390/ijms22126487 (PMC8234132; doi:10.3390/ijms22126487)
Supplement: Supplementary file 1 [file ijms-22-06487-s001.zip › ijms-1206174-supplementary.pdf]

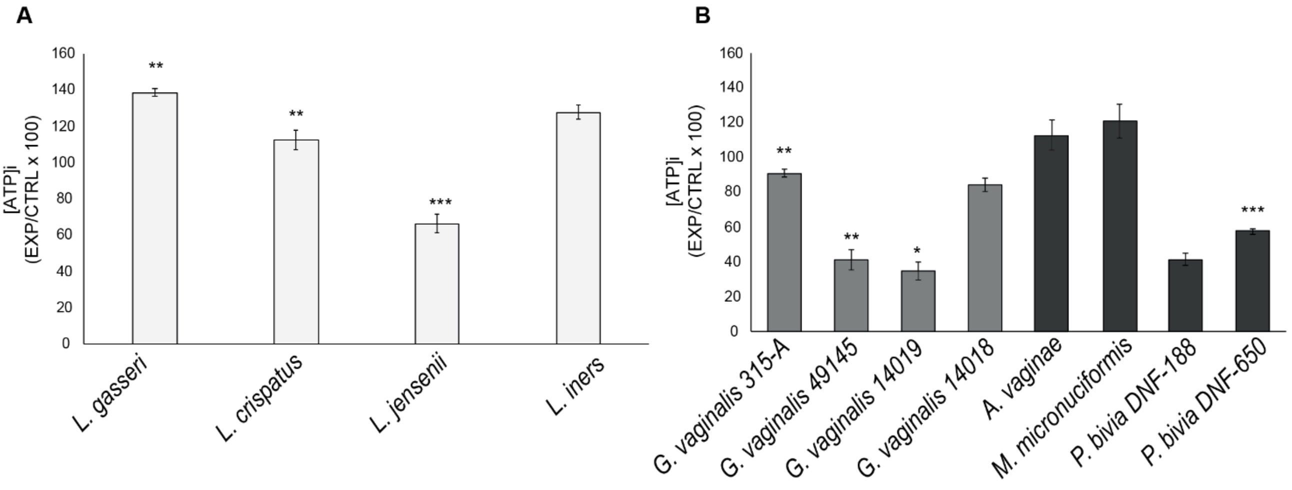

Supplementary figure S1. Effects of bacterial lysates on CaSki cell viability. ATP production by cells cultured with bacteria lysates (1µg/mL) from (**A**) *Lactobacillus spp.* or from (**B**) others vaginal dysbiosis bacteria (*A. vaginae*, *M. micronuciformis*, *P. bivia* DNF-188 and *P.bivia* DNF 650) for 24 hrs. The bar-graph shows results from one representative experiment out of three performed. Data are expressed as ATP produced by stimulated cultures/unstimulated control ± standard deviation (EXP/CTRL×100). Differences among stimulated and unstimulated cultures were evaluated by Student t-test. P * = p-value < 0.05; **= p-value < 0.01; ***= p-value < 0.001.

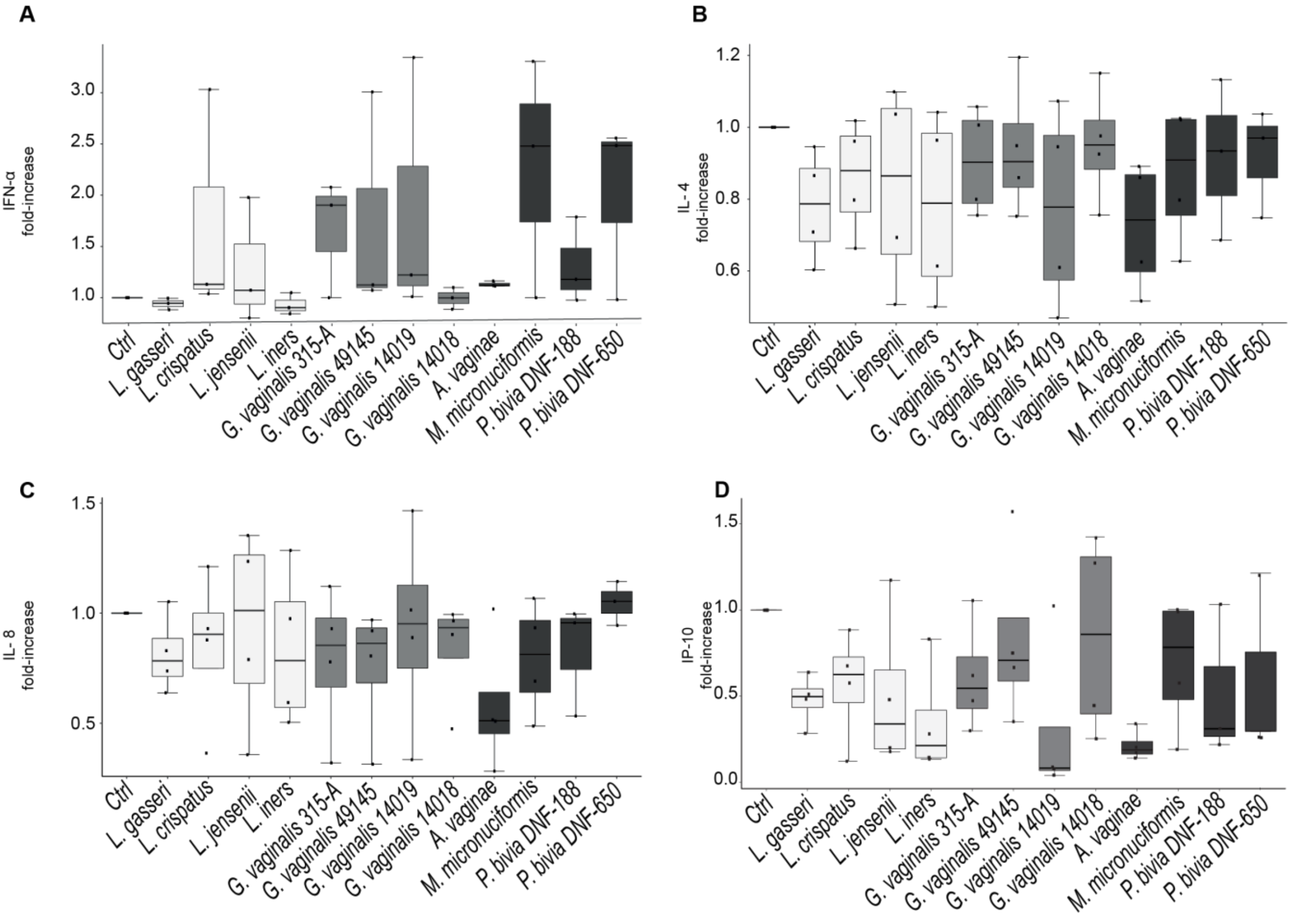

Supplementary figure S2. Cytokine's production by PBMCs stimulated with dominant species of vaginal microbiota. Cytokine concentration was measured in culture supernatants of bacterial-stimulated cultures. Results are expressed as fold increase in cytokine concentration respect to unstimulated cultures. The bar-graph shows the median and the whisker is calculated on the formula $IQR \times 1.5$. Statistical analysis was performed by Kruskal-Wallis. No significant differences among cultures were found.

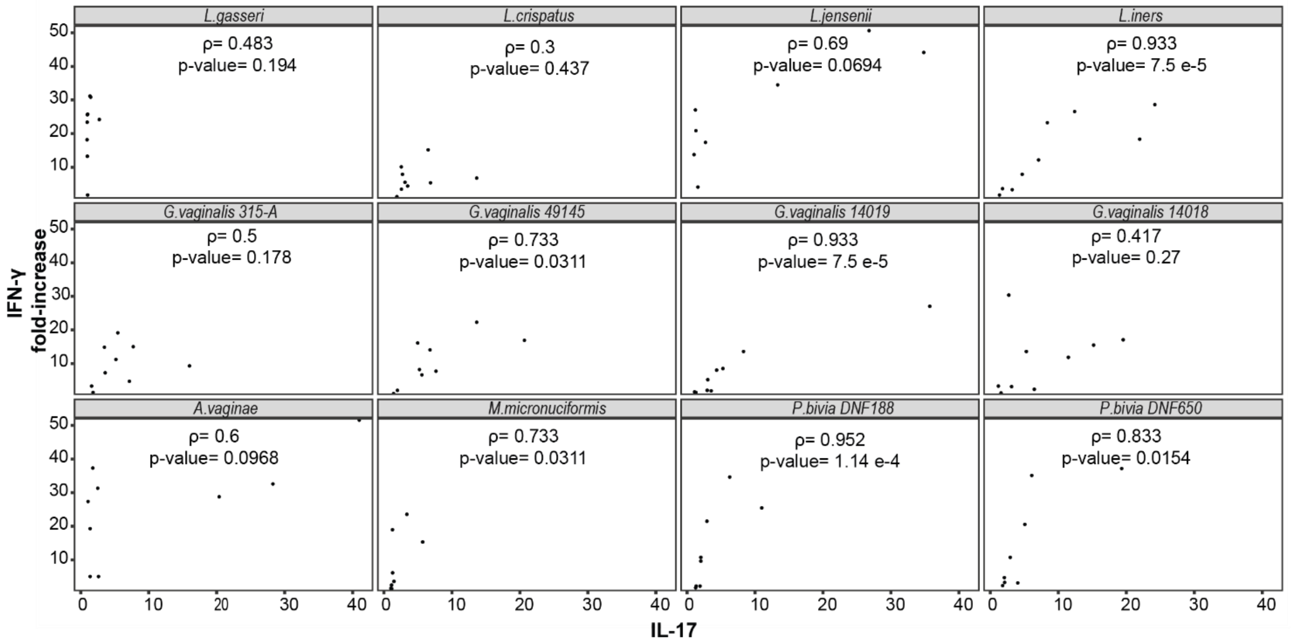

Supplementary figure S3. Spearman's correlation analysis among the cytokines' production induced by *Lactobacillus spp*. and other bacteria related to vaginal dysbiosis. Correlation among cytokines produced under different stimulation was evaluated by Spearman rank correlation analysis.

A

	IL-1β (pg/mL) ± SD	TNF-α (pg/mL) ± SD	IL-8 (pg/mL) ± SD
Ctrl	1.47 ± 0.13	4.01 ± 0.12	742.32 ± 8.41
L.gasseri	1.97 ±0.81	2.46 ± 0.11	647.05 ± 131.71
L.crispatus	1.26 ± 0.14	5.94 ± 0.08	568.69 ± 95.32
L.jensenii	1.22 ± 0.10	2.94 ± 0.13	707.51 ± 12.43
L.iners	1.15 ± 0.10	2.44 ± 0.09	739.50 ± 40.47
G.vaginalis 315-A	1.10 ± 0.09	6.60 ± 0.25	632.30 ± 52.15
G.vagnalis 49145	1.08 ± 0.03	1.71 ± 0.24	727.94 ± 27.74
G.vaginalis 14019	1.26 ± 0.07	7.75 ± 0.11	797.51 ± 13.256
G.vagnalis 14018	1.87 ± 0.28	7.02 ± 0.12	876.77 ± 17.57
A.vaginae	1.39 ± 0.28	2.97 ± 0.12	744.07 ± 49.36
M.micronuciforms	1.27 ± 0.30	7.48 ± 0.33	778.40 ± 10.28
P.bivia DNF188	1.08 ± 0.03	1.97 ± 0.17	740.15 ± 63.77
P.bivia DNF650	1.18 ± 0.05	2.43 ± 0.15	769.81 ± 3.75

B

	IL-1β (pg/mL) ± SD	TNF-α (pg/mL) ± SD	IL-8 (pg/mL) ± SD
Ctrl	1.90 ± 0.04	16.41 ± 2.29	2684.12 ± 106.32
L.gasseri	2.52 ±0.48	10.93 ± 1.44	2272.98 ± 51.08
L.crispatus	2.43 ± 0.64	8.99 ± 0.71	1953.39 ± 51.26
L.jensenii	2.61 ± 0.42	11.10 ± 1.11	2538.76 ± 82.63
L.iners	2.45 ± 0.35	10.00 ± 0.61	2848.66 ± 80.62
G.vaginalis 315-A	2.40 ± 0.14	8.88 ± 0.96	2176.82 ± 155.30
G.vagnalis 49145	2.43 ± 1.21	8.99 ± 0.95	2609.90 ± 113.51
G.vaginalis 14019	2.71 ± 0.11	9.45 ± 0.31	3182.85 ± 99.00
G.vagnalis 14018	2.61 ± 0.40	9.64 ± 0.21	2937.82 ± 177.05
A.vaginae	2.26 ± 0.14	8.52 ± 0.47	2877.37 ± 49.34
M.micronuciforms	3.09 ± 0.55	11.84 ± 0.95	2882.99 ± 169.91
P.bivia DNF188	2.22 ± 0.118	8.35 ± 0.39	2142.71 ± 70.51
P.bivia DNF650	2.30 ± 0.35	9.79 ± 0.74	1679.37 ± 102.46

Supplementary Table S1. Cytokine's production by epithelial cells stimulated with dominant species of vaginal microbiota. SiHa (**A**) and CaSki (**B**) cells were cultured with heat-inactivated bacterial cells (50 CFU/cell) from *Lactobacillus spp*., or *Gardnerella vaginalis*, or other vaginal dysbiosis bacteria (*A. vaginae*, *M. micronuciformis*, *P. bivia* DNF-188 and *P. bivia* DNF-650). Cytokine's concentration was assessed in supernatants after 5 days of culture through Multiplex Assay (IL-1β, TNF-α) or ELISA Assay (IL-8).
